# Identifying critical features of type two diabetes prevention interventions: A Delphi study with key stakeholders

**DOI:** 10.1371/journal.pone.0255625

**Published:** 2021-08-05

**Authors:** Jillian C. Ryan, Bonnie Wiggins, Sarah Edney, Grant D. Brinkworth, Natalie D. Luscombe-March, Kristin V. Carson-Chahhoud, Pennie J. Taylor, Annemien A. Haveman-Nies, David N. Cox

**Affiliations:** 1 Precision Health Future Science Platform, Commonwealth Scientific and Industrial Research Organisation, Adelaide, South Australia; 2 Public Health and Wellbeing Research Group, Commonwealth Scientific and Industrial Research Organisation, Adelaide, South Australia; 3 Physical Activity and Nutrition Determinants in Asia (PANDA), Saw Swee Hock School of Public Health, National University of Singapore, Singapore, Singapore; 4 Clinical Substantiation Group, Commonwealth Scientific and Industrial Research Organisation, Adelaide, South Australia; 5 Australian Centre for Precision Health, University of South Australia, Adelaide, Australia; 6 Chair group Consumption and Healthy Lifestyles, Wageningen University and Research, Wageningen, The Netherlands; Universiteit Twente, NETHERLANDS

## Abstract

**Aims:**

This study aims to identify critically important features of digital type two diabetes mellitus (T2DM) prevention interventions.

**Methods:**

A stakeholder mapping exercise was undertaken to identify key end-user and professional stakeholders, followed by a three-round Delphi procedure to generate and evaluate evidence statements related to the critical elements of digital T2DM prevention interventions in terms of product (intervention), price (funding models/financial cost), place (distribution/delivery channels), and promotion (target audiences).

**Results:**

End-user (n = 38) and professional (n = 38) stakeholders including patients, dietitians, credentialed diabetes educators, nurses, medical doctors, research scientists, and exercise physiologists participated in the Delphi study. Fifty-two critical intervention characteristics were identified. Future interventions should address diet, physical activity, mental health (e.g. stress, diabetes-related distress), and functional health literacy, while advancing behaviour change support. Programs should be delivered digitally or used multiple delivery modes, target a range of population subgroups including children, and be based on collaborative efforts between national and local and government and non-government funded organisations.

**Conclusions:**

Our findings highlight strong support for digital health to address T2DM in Australia and identify future directions for T2DM prevention interventions. The study also demonstrates the feasibility and value of stakeholder-led intervention development processes.

## Background

Growing prevalence of lifestyle-related chronic diseases including Type 2 Diabetes Mellitus (T2DM) has created an enormous need for innovative and effective ways to support people to proactively manage their health [1, 2]. T2DM directly affects about 1 million Australian adults, while a further 2 million are estimated to have pre-diabetes, indicating that they are at significant risk of developing T2DM in the near future [3]. While T2DM can be prevented or reversed by addressing its lifestyle-based antecedents, primarily, overweight or obesity, poor diet, and physical inactivity [4], translating this advice into actionable and effective behaviour change support is an ongoing public health challenge. Health-related behaviours are difficult to change and furthermore, sustaining health behaviour change is even harder, with behavioural changes often reverting to baseline levels over time [5].

Health interventions that harness digital delivery and technologies, such as smartphone apps and wearable trackers, are increasingly used to deliver health behaviour change support for people at risk or with T2DM and other chronic diseases [6]. Such digital approaches offer significant benefits. For example, digital interventions are able to integrate principles of ‘persuasive design’ such as personalisation, gamification, and social influence; as well as behaviour change techniques such as self-monitoring to encourage users to take up behavioural change [7–9]. They also piggyback on existing habitual smartphone and internet use to deliver intense behaviour change support programs. Furthermore, digital health interventions are highly scalable, able to be disseminated to large audiences at minimal cost-per-user, which is of critical importance in context of the growing need for such tools [10]. Taken together, digital health offers enormous potential as a cost-effective means to expand access to care among populations with access to digital technologies which may help to meet some of the growing need for chronic disease support and prevention [6].

Research and development related to digital health solutions for chronic disease is rapidly expanding. Interventions draw upon a wide range of health information technologies, including smartphone apps, intelligent algorithms (e.g. artificial intelligence or AI), continuous glucose monitoring, social media, and the internet more broadly [11]. They may be delivered via websites, smartphones, or text messages, and often seek to supplement clinical care options (e.g. telehealth) or change behaviours through self-monitoring of diet, activity, or glucose levels or through personalised health advice generated using AI [11]. To date, research has generated promising evidence for the efficacy of digital health interventions in controlled trial settings [12]. For example, one study by Spring and colleagues found an m-health intervention targeting multiple behavioural health risk factors was effective in improving activity and diet behaviours to recommended levels, with effects sustained to a 9-month follow up [13]. Digital translations based on the landmark Diabetes Prevention Program intervention [4] have also been shown to be effective in clinical trial conditions, with results sustained for several years in some cases [14, 15].

On the other hand, less is understood about how to best translate digital health solutions into real-world conditions and in ways that engage and meet the needs of diverse stakeholders [6]. Translating and implementing health evidence involves the design of programs that are highly acceptable, effective, and relevant to key stakeholders, including end-user and clinical stakeholders [16]. Achieving this is inhibited by a key limitation of digital health, which is that it is more easily abandoned by the user [17, 18]. Research by Vaghefi and Tulu identified a number of factors that drive abandonment of m-health programs, which included users’ perceptions of design elements (e.g. interface, navigation, and notifications), the depth of knowledge or content available in the app, and clarity of system rules [19]. Furthermore, changes in the individuals’ motivation and persistence also played an important role, with these factors tending to wane over time, leading to abandonment or reduced engagement with the program [19]. From the perspective of clinicians, factors affecting engagement with mobile health (m-health) tools relate more to the usability of such tools including interoperability with current work systems, as well as the influence of peers and whether tools are championed by others and the organisations themselves [20]. The range and complexity of factors affecting engagement with m-health highlight the need for stakeholder engagement in their design and evaluation. These challenges represent the next frontier for digital health, as we move towards the implementation of digital health for chronic disease into policy and practice [21].

Since effective implementation of health evidence is reliant on active support from the target audience, the broad inclusion of relevant stakeholders in multiple stages of research development and translation (i.e. participatory research) can increase the implementation potential of health interventions [22]. As a result, participatory research practices are strongly advocated by leading health authorities as a best-practice approach to evidence translation and implementation [23, 24]. Although full integration of participatory practices into health research is still limited, some studies have highlighted a range of benefits to service provision (e.g. shifts in organisational change, achieving collaboration and mutual learning, capacity building) [20] and translation outcomes [25, 26]. These include more novel and innovative idea generation and creativity and more support and enthusiasm for innovation [27]. Risks associated with failing to engage stakeholders have also been documented, which is considered to be a contributing factor towards high rates of research ‘waste’, including an estimated 50% of clinical trial results being unpublished and duplication of studies including the perpetuation of avoidable design flaws [28]. Participatory research practices can help to address challenges associated with engaging and retaining users and are therefore critical to progressing the impact of digital health into practice and policy [22].

In this participatory study, we worked with key stakeholders to identify key characteristics and needs associated with digital T2DM prevention and self-management solutions in the Australian context. We describe the process of identifying key stakeholders and engaging them in the initial steps of co-generating intervention ideas through a Delphi consensus study. Because T2DM is a serious medical condition that requires clinical support, we placed strong emphasis on the views of both end-user stakeholders (i.e. patients or those at risk of or diagnosed with T2DM) and professional stakeholders (i.e. health practitioners, T2DM managers, scientists and researchers, etc). This study represents the first step in a broader program of research that seeks to demonstrate how co-design can be used to develop interventions and translate them into community and practice. More specifically, the study aims to:

**Stage 1 Stakeholder Mapping**. Examine who are the key stakeholders for T2DM prevention and management in Australia and how can they be identified and recruited.
**Stage 2 Delphi Study**. Generate innovative ideas and identify key characteristics of future digital health interventions designed to help people with T2DM or pre-diabetes to adopt and sustain health behaviour change.

## Methods

This study was approved by the Commonwealth Scientific and Industrial Research Organisation Human Research Ethics Committee (Application #CSIRO_2019_102_LR) and conducted and reported in accordance with Guidance on Conducting and Reporting Delphi Studies [29]. All participants provided informed consent to take part in the study via the online survey. The study took place between September 2019 and June 2020.

### Stage 1. Stakeholder mapping and engagement

**Fig 1:**
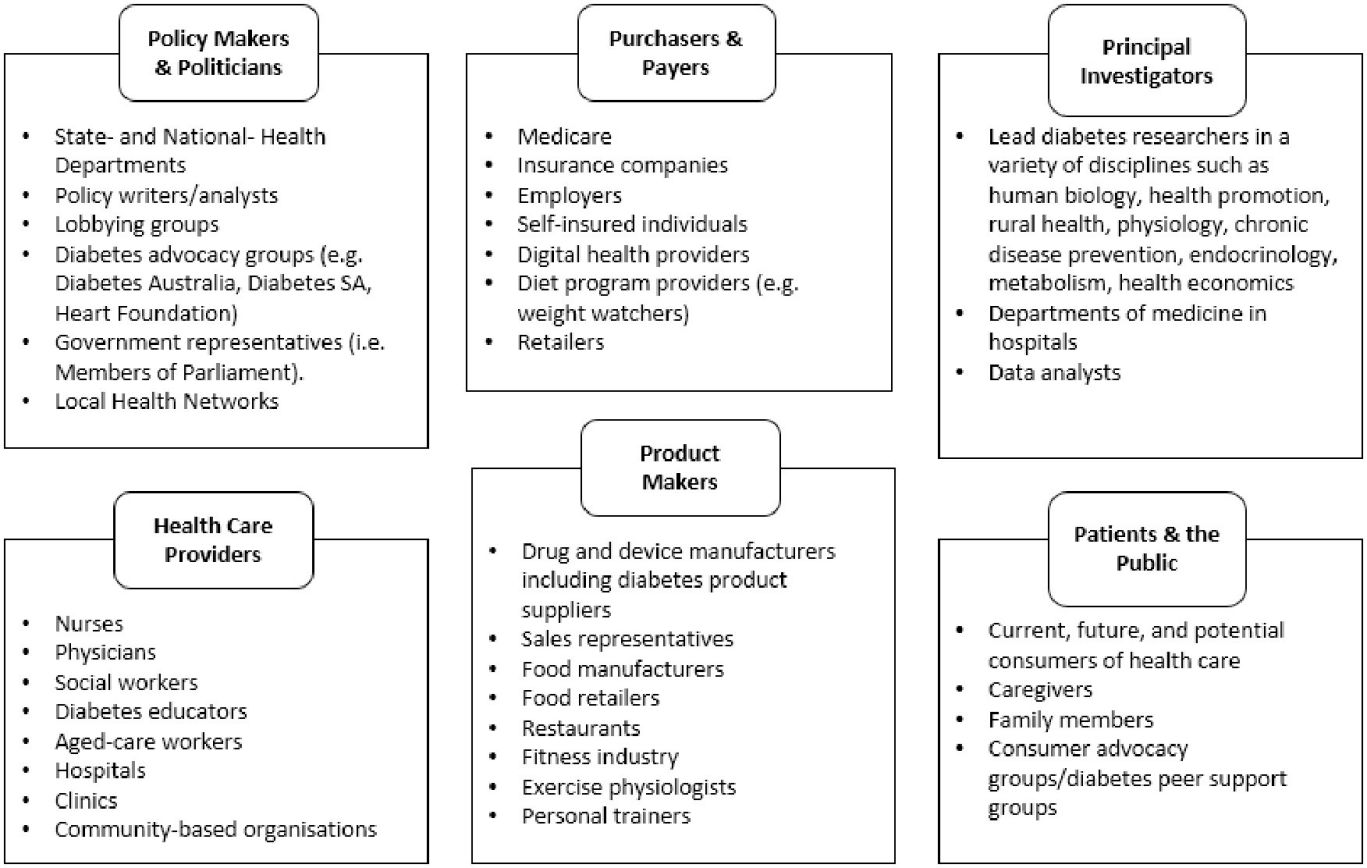
Stakeholder map including examples of key individual or organisation stakeholder roles.

We followed the stakeholder mapping process described by Schiller et al. [30] in order to identify and recruit a panel of relevant stakeholders. First, two members of the authorship team (J.R. and B.W.) created a list of disciplines relevant to T2DM prevention by collating the academic affiliations of authors and MESH headings of studies included in key relevant systematic reviews [31–34]. Once relevant academic disciplines and topics had been identified, these were extrapolated into professional, clinical, and policy-related roles, following Concannon et al.’s health stakeholder taxonomy structure (see Fig 1); Patients and the Public, Providers, Purchasers, Payers, Policy Makers, Product Makers, and Principal Investigators [35].

Relative importance of each stakeholder was then evaluated based on the perceived likelihood of interest (i.e. how determined an individual in this role is to address T2DM) and perceived strength of influence [30]. Each study author separately rated each stakeholder-type based on these criteria (interested/influential: yes/no) and then came together to discuss which stakeholder-types were of greatest priority.

#### Stakeholder engagement

Next, we sought to recruit stakeholders as Delphi study participants. Previous Delphi studies have reported a median of 17 (IQR: 11–31) participants, typically representing 2–3 stakeholder groups [36]. Smaller panels are vulnerable to significant influence from any one panel member and it is important to consider the weight of each respondents’ influence against the criteria for consensus (e.g. 80% must agree, in a panel of ten, one person = 10% of consensus) [37]. Anticipating dropout rates of approximately 20% over three rounds [38], we therefore aimed to recruit 76 stakeholders to ensure we would maintain the minimum sample size required for the study. We targeted two stakeholder categories, end-users (e.g. people at risk of T2DM or diagnosed with pre-diabetes or T2DM) and professional stakeholders (e.g. health practitioners, managers, scientists, etc with at least two years’ experience working in a diabetes-related field). Potential stakeholders were contacted via personalised emails that contained an invitation to take part and a weblink to an online registration survey. All stakeholders were offered an honorarium of $50 AUD upon completion of all three rounds of data collection.

To develop the list of professional stakeholder contacts, a template was prepared outlining all key stakeholder categories and types/roles. Authors were invited to populate the template based on their experience, which was supplemented by online searches as well as searches of the author list of relevant systematic reviews to create a broad list of stakeholders. End-user stakeholders were recruited from a database of previous research participants who had indicated that they would be interested in participating in future studies. To be eligible, end-user stakeholders had to self-identify as having T2DM or pre-diabetes. On the other hand, professional stakeholders were required to have at least two years’ experience working in a diabetes-related role but were not restricted based on any health-related criteria. All eligible participants were at least 18 years old.

#### Stakeholders

The stakeholder mapping procedure identified 171 potential participants (121 professional and 50 end-user stakeholders) who were eligible to participate in the Delphi study. A weblink to an initial Expression of Interest (EOI) to participate was emailed to all 171 stakeholders. In total, 112 stakeholders clicked on the EOI and of these, 76 completed the study enrolment procedure and Round One survey instrument (68% acceptance rate). Of these, 61 stakeholders participated in the three Delphi rounds (80% completion rate).

### Stage 3: Stakeholder consultation via Delphi study

Stakeholders were consulted on their perceptions of the ideal characteristics of future digital health programs via a three-round classical Delphi study (Fig 3). Delphi studies are a systematic approach to achieving consensus among key stakeholders, to gain insight or clarity in an area of challenge or complexity. The Delphi study was administered via online survey using Survey Gizmo software and each round had a pre-determined and specific purpose (described subsequently). Prior to commencing each survey, stakeholders were instructed to read context-setting information explaining the purpose of the study and to provide definition of key terminology such as pre-diabetes.

#### Delphi Round One

The purpose of the Delphi Round One was to generate evidence statements about preferred characteristics of digital T2DM interventions. Via an online survey, participants answered open-ended questions related to the marketing mix (i.e. product, price, place, and promotion, [39]), for example, ‘how should (a digital health T2DM program) be delivered?’. No pre-specified ideas were provided to participants. These were followed by nine questions to capture the respondents’ age, sex, education level; employment status, occupation, and income; diabetes status, and contact information.

Round 1 data was analysed using inductive thematic analysis to group responses in themes [40]. First the data were exported into Microsoft Excel. Following data familiarisation, one author analysed the data by placing each response into broad themes. These categorisations were validated by another author to establish inter-rater reliability. Finally, the remainder of the dataset was categorised, and the evidence statements were then presented to the broader research team for feedback and refinement.

#### Round Two

In Round Two stakeholders rated each of the themes or evidence statements generated in Round One using the 9-point Grading of Recommendations, Assessment, Development and Evaluations (GRADE) scale with three categories: not important (1–3), important but not critical (4–6), and critically important (7–9) [41]. Stakeholders were also able to suggest new items or comment on existing items in Round Two.

*Definition of consensus*. Round Two data were analysed descriptively to determine which evidence statements reached consensus. Evidence statements that reached consensus were automatically passed through to the final round while statements that did not reach consensus were passed through to Round Three for further evaluation. Statements were deemed to have reached consensus when a certain level of group agreement was reached, more specifically, at least 70% of respondents rated an item as ‘critically important’ *and* no more than 15% of respondents rated that same item as ‘not important’ [42]. Conversely, in the instance that at least 70% of respondents rated an item as ‘not important’ and no more than 15% rated the item as ‘critically important’, items could be deemed unimportant by consensus. Responses and the level of agreement for each statement were analysed separately for the two stakeholder groups.

#### Round Three

**Fig 2:**
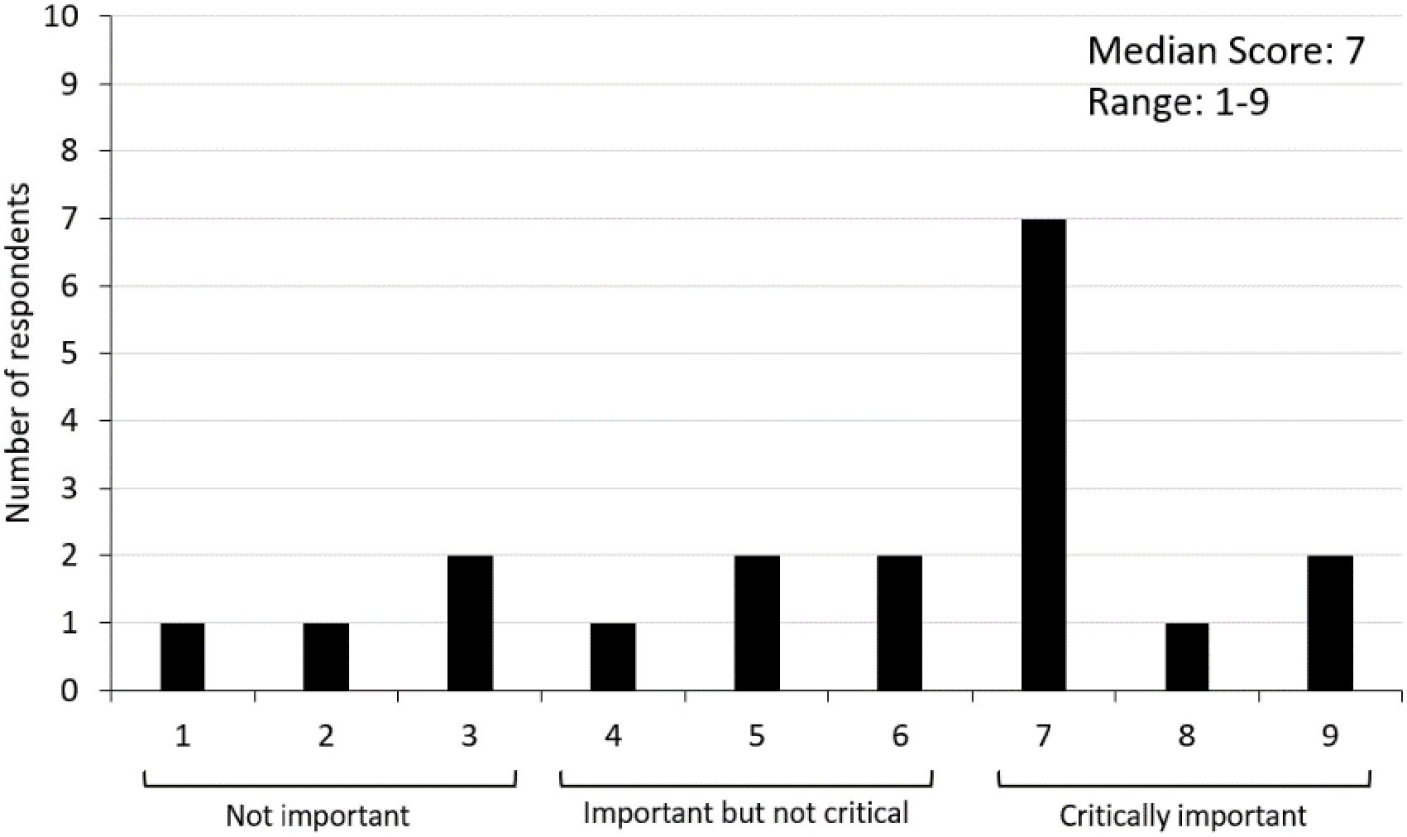
Example of group-level feedback on ratings of one evidence statement capturing the importance of supermarket tours as an intervention component.

The aim of Round Three was to provide an opportunity for participants to re-rate statements that did not reach consensus with the objective to increase group consensus about its importance (while recognising that only a small increase in consensus is likely) and to further clarify respondents’ appraisal and importance of each statement [43]. Stakeholders were presented with the statements that had not reached consensus in Round Two as well as additional information about how their participant group (end-user or professional stakeholders) rated each statement. More specifically, the median and range scores and a frequency histogram depicting the spread of scores were provided. An example presented in Fig 2 displays professionals’ ratings of the importance of including supermarket tours in digital T2DM programs (a ‘product’ variable).

#### Flow of evidence statements throughout study

**Fig 3:**
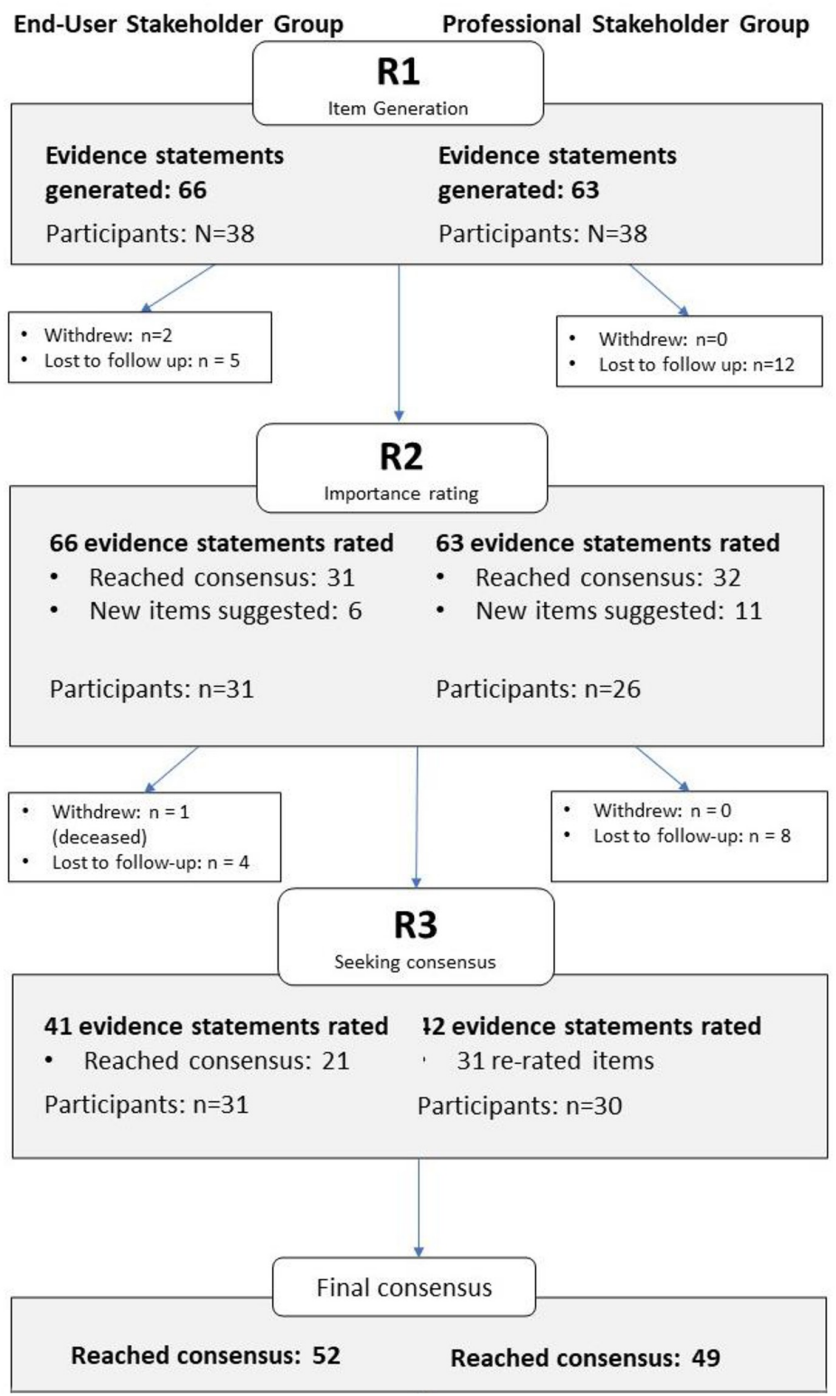
Participant and data item flowchart.

Round One identified 66 evidence statements (end-user stakeholders) and 63 statements (professional stakeholders). The flow of evidence statements through Rounds One to Three is depicted in Fig 3. By Round Three, 52 (end-user stakeholders) and 49 (professional stakeholders) statements met group consensus of critical importance while no statements in either group met the criteria for consensus of non-importance.

## Results

### Participants

A total of 76 stakeholders enrolled in the study. Of these, 38 were end-user stakeholders, 32% of whom were female and 84% of whom were at least 50 years old (see Table 1). Twenty-four percent of the end-user stakeholder group had pre-diabetes while 63% had T2DM and 13% were not sure whether they currently had T2DM or pre-diabetes. In the professional stakeholder group, 76% were female and 47% were at least 50 years old. Occupations of the stakeholders in the scientific/clinical stakeholders group included dietitians (n = 9, 23.7%) managers in relevant organisations (e.g. CEO; n = 6, 15.8%), credentialed diabetes educators (n = 4, 10.5%), data analysts (3, 7.9%), nurses (n = 4, 10.5%), medical doctors/physicians (n = 4, 10.5%), research scientists (n = 4, 10.5%), social workers (n = 2, 5.3%), an exercise physiologist (n = 1, 2.6%), and a pharmacist (n = 1, 2.6%).

**Table 1:** Participant characteristics.

	End-user stakeholders (N = 38)	Professional stakeholders (N = 38)
Gender (n, %n female)	12 (31.6%)	29 (76.3%)
Age (n, %n)		
20–29	-	4 (10.5%)
30–39	3 (7.9%)	9 (23.7%)
40–49	3 (7.9%)	7 (18.4%)
50–59	17 (44.7%)	7 (18.4%)
60–69	15 (39.5%)	6 (15.8%)
Missing	-	5 (13.2%)
Highest level of education attained (n, %n)		
Year 11 or below	6 (15.8%)	-
Year 12 or equivalent	4 (10.5%)	-
Vocational training	10 (26.3%)	1 (2.6%)
University degree	12 (31.6%)	9 (23.7%)
Postgraduate degree	6 (15.8%)	26 (68.4%)
Missing		2 (5.2%)
Self-reported T2DM status		
Current T2DM diagnosis (n, %n yes)	24 (63.2%)	0
Current pre-diabetes diagnosis (n, %n yes)	9 (23.7%)	1 (3.8%)
Unsure of current diagnosis	5 (13.1%)	1 (3.8%)

### Product

The product variable considers what features digital T2DM health interventions should include. Features that promote positive change in the areas of physical activity, diet, mental health (e.g. stress, diabetes-related distress), health literacy, access to health care services, and behaviour change success reached consensus agreement of critical importance in both participant groups (see Table 2). Several additional statements reached consensus agreement of importance in only one of the participant groups. By consensus, end-user stakeholders reported meal plans as critically important to achieving diet change and reported additional funding to cover the cost of medical treatment of critical importance while scientific/clinical stakeholders did not.

**Table 2:** Participant-generated ideas for health promotion program.

Intervention domain	Evidence statement	Rated as critically important by:	
		End user stakeholders	Professional stakeholders
Physical activity	• Education about the link between physical activity and T2DM	✓	✓
	• Practical resources to support physical activity (e.g. subsidised gym memberships, exercise programs.)	✓	✓
	• Exposure to relevant allied health care scientific/clinical stakeholders (e.g. exercise physiologists).	✓	✓
Diet	• Education about the link between diet and T2D	✓	✓
	• Healthy recipes	✓	✓
	• Meal plans	✓	✗
	• Access to fresh and healthy foods (e.g. affordable fruit and vegetables)	✓	✓
Psychological health	• Education about the link between T2D and mental health	✓	✓
	• Stress management techniques	✓	✓
	• Positive mental health promotion	✓	✓
Health literacy	• Education about the links between health and lifestyle factors (e.g. smoking, physical activity, diet, alcohol).	✓	✓
	• Education about the link between T2D and cardio-vascular disease.	✓	✓
	• Demographic health risk factors (e.g. sex, age)	✗	✓
Access to health care services	• Funding/subsidies to cover the cost of medication or medical treatment	✓	✗
	• Education about how to access funds to pay for medical costs (e.g. Medicare, private health insurance)	✓	✓
	• Contact/exposure to a variety of health care scientific/clinical stakeholders	✓	✓
Behaviour change	• Success stories to motivate and empower users	✓	✓
	• Reviews of current lifestyles to help identify areas for improvement.	✓	✓
	• Access to biomarker self-monitoring technology (e.g. continuous glucose monitoring).	✓	✓
	• Behavioural self-monitoring (e.g. exercise and diet diaries).	✓	✓
	• Online social support networks	✓	✓
Environmental strategies	• Changes to policy (e.g. sugar tax, free access to lifestyle programs)	✗	✓
	• Increase neighbourhood walk-ability (e.g. number of safe pathways that connect to important places like bus stops, shops)	✓	✓
	• Increase available green space in neighbourhood (e.g. parks, trees, gardens)	✗	✓

### Promotion (target audience)

Promotion considers the target audience of the product. End-user and professional stakeholders agreed that specific programs should be developed for the different target audiences, in particular.

- people with pre-diabetes or at high risk,
- people with T2DM,
- adults in general,
- families of people with diabetes, and
- people who live in regional, rural, or remote areas specifically.

Early intervention/health promotion for both children and adults were also identified as critically important by both participant groups.

### Place

Place considers where and how the product should be delivered. All groups agreed that programs should be delivered via digital platforms (e.g. website, mobile phone application) and/or via a mixture of delivery approaches, depending on context. In addition, professional, but not end-user stakeholders, considered it critically important that interventions have face-to-face components. Conversely, end-user stakeholders, but not professional stakeholders, considered it critically important that interventions are delivered via school-based programs.

### Price

Stakeholders were asked who should fund the provision of T2DM prevention programs. Both groups agreed that such programs should be funded by a combination of stakeholders, reflecting demand for co-funded arrangements that include input from both the Federal and State governments and the contribution of in-kind provision of evidence (e.g. by scientific organisations and universities) or program delivery (e.g. non-governmental organisations like Diabetes Australia). Furthermore, the end-user but not professional stakeholder group considered that programs should be funded by a range of other entities including not-for-profit organisations, universities, and stakeholders; while scientific/clinical stakeholders thought that local government and councils should fund such programs.

## Discussion

This study used stakeholder mapping combined with a three-round Delphi study to identify critically important characteristics of digital T2DM prevention interventions. The Delphi consultation process identified at least 52 characteristics considered critically important by consensus within the two groups of stakeholders. Key findings include firstly, that digital tools are needed to help the target population to improve their physical activity levels, diet, mental health, and functional health literacy, which includes their ability to manage their health and knowledge of health care systems. Secondly, programs are needed that incorporate behaviour change techniques that seek to increase self-efficacy for change, as well as measures to address environmental limitations on health behaviours (e.g. lack of walking paths). Thirdly, it is critically important that programs target people who are at-risk of T2DM before they develop T2DM (primary prevention). In addition, vulnerable groups such as those who live in rural and regional remote areas require tailored programs that differ from those targeting the general population. Finally, programs are needed that are entirely or partially delivered via digital means.

### Comparison with previous literature

Similar to previous research [44] this study uncovered some novel findings while also confirming some commonly-accepted arguments within chronic disease prevention. Our finding that future T2DM prevention programs should target physical activity, diet, psychological health, health literacy, access to healthcare services and behavioural change was consistent with a similar study conducted in the UK. In that research, participants living with T2DM sought assistance with diet and physical activity, support for self-management strategies, assistance in understanding in-depth or complex information in an intervention [44].

A strong theme that ran through participant responses was the need for tools that help to boost health literacy in patients with pre-diabetes. Previous research has similarly highlighted uncertainty associated with the diagnosis of pre-diabetes and what was going to happen to patients as a major source of stress for people with pre-diabetes [45]. Although health literacy is a well-known determinant of health and predictor of chronic diseases, conventional operationalisations of health literacy within diabetes interventions have had a strong focus on health education about disease self-management and lifestyle factors [46] as well as health literacy and numeracy [47], without addressing the broader contextual, practical, and functional aspects of health literacy identified in this study, which are also part of more contemporary health literacy frameworks [48]. Functional health literacy, as described by Osborne and colleagues, contains nine conceptually distinct areas of health literacy, such as ‘having sufficient information to manage my health’, ’feeling understood and supported by healthcare providers’, and ’ability to actively engage with healthcare providers’ [48]. Educational interventions are essential to patients’ ability to seek further support, understand the health care system, and take steps to change their lifestyles, which have significant implications for health outcomes.

In Australia, pre-diabetes is often approached in a watch-and-wait approach with limited health behaviour change support offered, which is contributing to high rates of progression to T2DM. A limited set of national and state-based T2DM education tools are available, many of which are based upon evidence-based interventions such as the DESMOND model [46]. The National Diabetes Services Scheme (NDSS), for example, offers a ‘Type 2 Diabetes and Me’ free online course (https://www.ndss.com.au/services/support-programs/), as well as support programs and information sessions. However, few of these programs are specific to pre-diabetes and furthermore it is not clear or visible to the end-user stakeholder whether current offerings were developed on a foundation of meaningful community engagement, which can limit community uptake of such programs [49]. Community-driven initiatives that support the unique health literacy support needs of people with pre-diabetes and are visibly stakeholder-driven remain a promising area for research to improve the translational impact of T2DM prevention education initiatives.

In terms of program delivery, our results indicate that while professional stakeholders group considered face-to-face interactions (either group-based or one on one) as critically important, end-user stakeholders did not. This is interesting in comparison with previous research where adults with T2DM in Europe expressed a desire for interactive, face-to-face treatment and lifestyle support intervention [50]. It is possible that the COVID-19 pandemic has accelerated clinical implementation and subsequent adoption and acceptance of e-health and telephone clinical services, which may have also contributed to high demand for digital services detected in our study. In the future, digital and face-to-face clinical services need to co-exist in blended models as one is not a complete solution.

### Stakeholder engagement: Lessons learned

Study acceptance and completion rates were high, both from a practical perspective (80% of participants completed the study to the end) and relative to previous Delphi studies [36, 51]. As this process was delivered via an entirely hands-off and online protocol the study demonstrates an efficient and potentially cost-effective method of meaningfully engaging with stakeholders in the co-design of digital health for chronic disease interventions [36, 51]. Similar to previous research [51], anecdotally it was noted that the use of personalised contact including phone calls to each participant likely contributed to the high-quality engagement achieved. A number of pragmatic benefits to participating in Delphi studies were also mentioned in participant communication. An honorarium that approached fair compensation for stakeholders’ time was appreciated, while it was reminded that participation in this type of research and stakeholder engagement activities can count towards continuing professional development points for certain roles. In future, these benefits might be communicated clearly in recruitment materials to facilitate participant recruitment and retention.

### Directions for future research

This study provides some direction on the design of digital health prevention programs for T2DM. Currently, few evidence-based, disease-specific programs are widely available in Australia to help people to adopt healthier lifestyles. Further research is needed to identify how evidence-based solutions can be effectively translated into the community and clinical practice. User-centred methodologies including Delphi studies and other co-design strategies will be key to overcoming the complex challenge of designing interventions that are appealing and engaging for end users.

Further research is needed to identify needs and preferences among under-served, minority, elderly, and other vulnerable groups. While our findings may be generalisable to the majority of the Australian population, they have also highlighted the need for unique solutions tailored towards people living in remote and rural areas, people from culturally and linguistically diverse backgrounds, and other under-served, minority, and vulnerable groups. In addition, as digital health continues to spread through our health systems, further work is needed to understand and address the growing ‘digital divide’ including who and in what ways people may be disadvantaged by the digital revolution. Digital health is evolving rapidly, with a stream of new technologies entering the market each year, making it difficult to keep abreast of the latest innovation. To maximise returns on investment, attention should focus on technologies that are succeeding in other fields, such as education or gaming, to identify overlaps for health. More research investment is needed in this area.

### Study strengths and limitations

This study applied strict adherence to the classical 3-round Delphi Study approach. This approach was effectively implemented and able to demonstrate the value of acquiring consensus in complex health issues, namely T2DM. Finally, this study sought the involvement of both professional stakeholders actively working in the field of T2DM, and potential end-user stakeholders of a T2DM prevention programme, and therefore gained insights from two distinct but equally relevant stakeholder groups. This extends previous work assessing a singular stakeholder group [44] and that enabled the analysis of consensus achieved in either groups, or as a total sample, and assisted in identifying similarities and differences what each group values in an intervention.

In addition to study strengths it is also important to consider the limitations. Study findings are based on a sample of stakeholders and may not necessarily be representative of all end-user or professional stakeholders’ views. While thorough consideration was given to the selection of stakeholder groups, it is possible that some key professions were underrepresented in the professional stakeholder group. For example, it was noted during data analysis that educators including teachers and those involved in establishing the school curriculum are relevant stakeholders, however, these were not considered in our stakeholder map and only emerged from the results. In terms of the socio-demographic profiles of stakeholders there is also a gender bias with end-user and professional stakeholder groups skewed towards males and females, respectively. Finally, the specific T2DM outcomes of this study are specific to the Australian health landscape as the content is specific to the Australian population and perhaps other countries with freely available universal healthcare, though the methodological contributions may have more general application.

## Conclusions

This 3-round classical Delphi study confirm the need for further evidence- and theory- based tools that support improvements in physical activity, diet, and mental health as core targets, as well as an expanded definition of functional health literacy that encompasses the skills and knowledge needed to access health care services. The broader field of public health would benefit from further use of stakeholder engagement through rigorous research designs, such as Delphi studies, to analyse complex and multi-faceted areas of health management and disease prevention.
